# Effects of Medicinal Leech-Related Cationic Antimicrobial Peptides on Human Blood Cells and Plasma

**DOI:** 10.3390/molecules27185848

**Published:** 2022-09-09

**Authors:** Tatyana V. Vakhrusheva, Grigoriy D. Moroz, Liliya Yu. Basyreva, Ekaterina V. Shmeleva, Sergey A. Gusev, Elena V. Mikhalchik, Ekaterina N. Grafskaia, Ivan A. Latsis, Oleg M. Panasenko, Vassili N. Lazarev

**Affiliations:** 1 Laboratory of Physical-Chemical Methods of Investigation and Analysis, Federal Research and Clinical Center of Physical-Chemical Medicine of Federal Medical Biological Agency, 119435 Moscow, Russia; 2 Department of Biological and Medical Physics, Moscow Institute of Physics and Technology (State University), 141701 Dolgoprudny, Russia; 3 Laboratory of Genetic Engineering, Federal Research and Clinical Center of Physical-Chemical Medicine of Federal Medical Biological Agency, 119435 Moscow, Russia

**Keywords:** antibacterial agents, synthetic cationic antimicrobial peptides, medicinal leech, cell membrane disruption, plasma coagulation

## Abstract

Cationic antimicrobial peptides (CAMPs) are considered as next-generation antibiotics with a lower probability of developing bacterial resistance. In view of potential clinical use, studies on CAMP biocompatibility are important. This work aimed to evaluate the behavior of synthetic short CAMPs (designed using bioinformatic analysis of the medicinal leech genome and microbiome) in direct contact with blood cells and plasma. Eight CAMPs were included in the study. Hemolysis and lactate dehydrogenase assays showed that the potency to disrupt erythrocyte, neutrophil and mononuclear cell membranes descended in the order pept_1 > pept_3 ~ pept_5 > pept_2 ~ pept_4. Pept_3 caused both cell lysis and aggregation. Blood plasma and albumin inhibited the CAMP-induced hemolysis. The chemiluminescence method allowed the detection of pept_3-mediated neutrophil activation. In plasma coagulation assays, pept_3 prolonged the activated partial thromboplastin time (APTT) and prothrombin time (at 50 μM by 75% and 320%, respectively). Pept_3 was also capable of causing fibrinogen aggregation. Pept_6 prolonged APTT (at 50 μM by 115%). Pept_2 was found to combine higher bactericidal activity with lower effects on cells and coagulation. Our data emphasize the necessity of investigating CAMP interaction with plasma.

## 1. Introduction

The increasing emergence and spread of bacteria resistant to current antibiotics necessitate a search for new-generation antibacterial agents. Cationic antimicrobial peptides (CAMPs) could be among the appropriate compounds for this purpose [1,2,3,4]. Naturally occurring CAMPs are known to exert multiple activities including antibacterial, antiviral, antifungal, antiparasitic, and insecticidal ones [5]. CAMPs are synthesized in all living organisms from prokaryotes to human beings, and are a part of innate immunity [6].

CAMPs found in nature are generally composed of fifty or less amino acid residues and possess a net charge from +2 to +11 (at neutral pH) [7,8]. They are highly heterogeneous in amino acid sequences and secondary structures. It is believed that the mechanism of CAMP action on bacteria is such that it may reduce the development of resistance. In addition, it is "tuned" to the cell wall and membrane of bacteria. Bacterial membranes contain many anionic lipids in contrast to eukaryotic membranes containing zwitterionic lipids and cholesterol. CAMPs are electrostatically attracted to negatively charged bacterial surface molecules. After adsorption onto the cell surface, the peptide hydrophobic residues are inserted into membrane lipids. Two modes of CAMP action, membranolytic and non-membranolytic, are generally distinguished [9,10,11,12]. In the first one, CAMPs disrupt the membrane, which leads to cell lysis and death. In the second, CAMPs enter the cell without causing lysis and inhibit essential cell functions by binding to nucleic acids and proteins [13,14,15,16]. Since CAMPs non-specifically attack multiple low-affinity targets rather than one certain high-affinity target characteristic for traditional antibiotics, it is difficult for bacteria to defend themselves by developing a single resistance mechanism [12,17].

The selectivity of some CAMPs for bacterial surface over eukaryotic plasma membrane allows, among exogenous CAMPs (designed or derived from organisms), those that are much less toxic to humans rather than to bacterial cells to possibly be found and selected [18].

Though CAMPs have been widely and long discussed as potential anti-infectious therapeutics, only a few of them are currently approved for clinical use [19]. Among them are tyrocidine, gramicidin, polymyxins, and bacitracin, all being derived from bacilli. They are the oldest therapeutic CAMPs (gramicidin was approved by the FDA in 1955) and are still used today [20,21,22]. Another naturally occurring CAMP daptomycin (from *Streptomyces roseosporus*) has been introduced into clinical practice more recently (approved by the FDA in 2003). Slow progress in the development of CAMPs available for human medicine may be explained by a number of reasons. An important one is high production costs. However, new peptide manufacturing technologies incorporating chemical synthesis may probably improve this situation, and short peptides (less than thirty residues) in this case have an advantage due to the simple structure and, hence, cost-effectiveness [23,24,25]. There are also limitations such as CAMP instability in the human body and toxicity towards human cells. To date, no designed CAMP antimicrobials have been implemented into clinical practice yet. Nevertheless, trials of new CAMPs at the preclinical and clinical stages are under way [22,26].

The relative ease of chemical peptide synthesis in combination with engineering approaches has prompted scientific interest on these compounds. Prediction mechanisms based on machine learning have been developed to analyze natural CAMP sequences and determine the relevant characteristics to extend them for designing novel CAMPs [12,27,28]. Since synthesizing of CAMPs allows for systematic structural variation, it provides a way for structure–activity relationship studies to optimize these molecules, improving their antimicrobial effectiveness while reducing side effects [29,30,31,32].

Substantial advances have been made in the identification of novel CAMP sequences. However, even if CAMPs have high and broad-spectrum antimicrobial activity, their usefulness depends on other various factors, including side effects in the body. Cytotoxicity assessment as usually measured by hemolytic activity is apparently far from sufficient evaluation of CAMP biocompatibility. Cationic nature, hydrophobicity and amphipathicity, the molecular features important for CAMP’s antimicrobial activity, can promote binding to host cellular membranes as well as to non-cellular components (e.g., proteins, lipoproteins). To date, there is a very limited knowledge of CAMP action on cell types other than erythrocytes and of a full spectrum of possible host “targets” for CAMPs, in particular in blood.

This study utilized a number of short synthetic CAMPs we earlier constructed using bioinformatic analysis of the medicinal leech genome and microbiome. The CAMPs had diverse amino acid sequences, and included those with relatively low minimum inhibitory concentration (MIC) and those with relatively high MIC. Blood-sucking leeches as a source of CAMP candidates for medical applications are of interest, since they are able to keep ingested blood for a long time without degradation due to the leech’s anticoagulants [33,34] and CAMPs [35,36,37], which allows suggesting that leech’s CAMPs exert low toxicity to blood cells. Additionally, since leeches feed on the blood of vertebrates, it may be suggested that their CAMPs are adapted to microorganisms that are host pathogens, in particular mammal pathogens. The present study is devoted to search for various possible effects of CAMPs in human blood. At the same time, it was aimed to compare our novel CAMPs regarding biocompatibility aspects. Beyond a hemolysis test, which is often used alone to estimate the cytotoxicity of CAMPs, the cell-lysing activity of CAMPs was determined with mononuclear cells and neutrophils, the two most abundant leukocyte subpopulations. Hemolysis was assayed in the absence or presence of blood plasma or albumin to elucidate how they affect the cytolytic activity of CAMPs. It was also examined whether CAMP-mediated neutrophil activation can occur. Furthermore, the influence of CAMPs on plasma coagulation was investigated. The results obtained gave an estimation of the ability of CAMPs under study to damage blood cell plasma membranes, and suggest a difference in susceptibility to CAMP action between different cell types. Blood plasma and its major constituent albumin inhibited CAMP-induced hemolysis, suggesting CAMP binding in plasma. However, despite high albumin concentration, other plasma proteins can compete with it for CAMPs, as shown by the anticoagulant effect of CAMPs, indicating their binding to coagulation factors. Protein binding of AMPs decreases their availability for bacteria, and at the same time, alters protein structure/function. One of the studied CAMPs exerted an ability to induce erythrocyte aggregation. In general, the data indicate a variety of possible behaviors of different CAMPs in contact with blood.

## 2. Results

### 2.1. Characteristics of CAMPs Used in this Study

CAMPs were designed and synthesized as described previously in our works [33,34,35]. Their amino acid sequences, molecular masses and charges are provided in Table 1. The minimum inhibitory concentrations (MICs) for the Gram-negative bacterium *Escherichia coli* and Gram-positive bacterium *Bacillus subtilis* were determined earlier or in the present study [38,39,40]. The MIC values for *Escherichia coli* were the same or higher than those for *Bacillus subtilis.* They are shown in Table 1. The CAMP concentrations tested were in the range up to 100 μM.

For additional characteristics describing physical–chemical properties of CAMPs, see Supplementary Table S1.

### 2.2. Effect of CAMPs on Plasma Membrane Integrity of Erythrocytes

Five CAMPs, pept_1, pept_2, pept_3, pept_4 and pept_5, were included in our cytotoxicity study. Hemolysis testing is a common initial stage in cytotoxicity assessment. The widely used hemolysis assay measures the amount of hemoglobin released through a damaged erythrocyte membrane into the extracellular medium. We applied the assay to carry out a comparative evaluation of hemolytic activity of our CAMPs and to elucidate whether blood plasma and albumin, the most abundant protein in plasma, could interfere with this activity.

Erythrocytes were incubated (0.5 h, 37 °C) with different CAMP concentrations ranging from 6.25 μM to 100 μM. Incubation conditions chosen were similar to those used in other studies [29,41,42]. Following incubation, the samples were centrifuged, and the supernatant analyzed spectrophotometrically for hemoglobin at 414 nm and 540 nm. Absorption at 414 nm provides higher sensitivity. The results were the same no matter which wavelength was used to monitor for hemoglobin.

**Figure 1:**
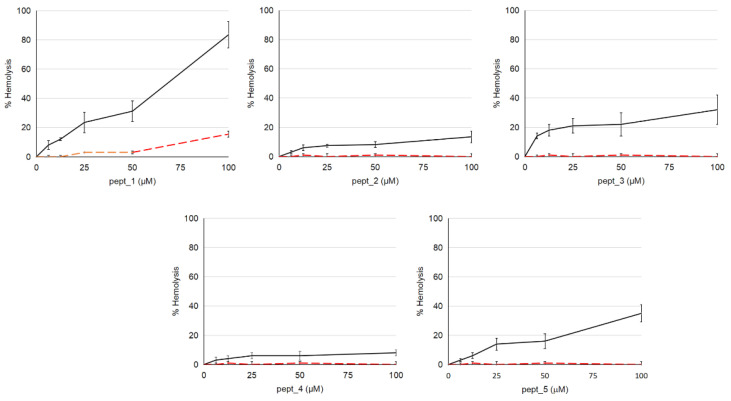
Hemolytic activity of the cationic antimicrobial peptides (CAMPs) pept_1, pept_2, pept_3, pept_4 and pept_5. Solid lines—erythrocytes were added to the same volume of PBS containing the peptide; dashed lines—erythrocytes were added to the same volume of PBS containing the peptide and autologous plasma. Peptide concentrations indicated on the X-axis are final concentrations in the cell-containing samples, with a final plasma dilution of 1:10. Hemolysis was assayed after 0.5 h of incubation at 37 °C. Cells with Triton X-100 served as a positive control, which was set to 100%. Data shown are mean ± SD from three independent experiments using blood from three subjects, with triplicate samples in each experiment.

Dose-dependencies of CAMP-induced hemolysis are presented in Figure 1. Pept_1 was found to have a higher hemolytic activity among the tested CAMPs, with an about 80% hemolysis at 100 μM. Pept_3 and pept_5 showed a medium hemolytic activity. They induced, at 100 μM, a 20–40% hemolysis. Pept_2 or pept_4 at the same concentration caused only a 10–15% hemolysis. Thus, according to their hemolytic potency, CAMPs could be arranged in the following descending order: pept_1 > pept_3 ~ pept_5 > pept_2 ~ pept_4. However, all these CAMPs were considerably less cytotoxic than the well-known lytic CAMP melittin from bee venom. Its 1 μM concentration caused 100% hemolysis.

**Figure 2:**
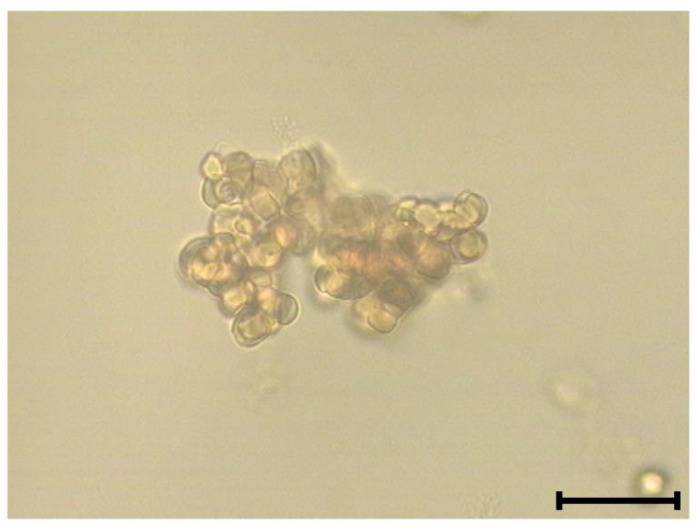
Representative image of an erythrocyte aggregate formed upon cell incubation in PBS containing the cationic antimicrobial peptide (CAMP) pept_3 (50 μM). Following incubation (0.5 h, 37 °C), cells were fixed with glutaraldehyde and photographed with a light microscope. Scale bar: 30 μm.

**Figure 3:**
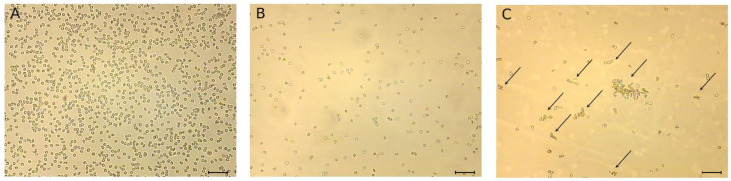
The cationic antimicrobial peptide (CAMP) pept_3 induces erythrocyte aggregation. Arrows indicate aggregated erythrocytes. Qualitative morphological evaluation of erythrocytes was performed using glutaraldehyde fixation method. Shown are light microscopy photographs of erythrocytes after incubation (0.5 h, 37 °C) in PBS with or without peptides, with a final peptide concentration of 50 μM. Control cell appearance (**A**); cell appearance after incubation with pept_1 (**B**) or pept_3 (**C**). Scale bar in each subfigure: 200 μm.

Pept_3 stood out from the other four CAMPs in that it not only lysed, but also aggregated cells. An erythrocyte aggregate formed is illustrated by Figure 2. Samples for morphological observation of erythrocytes were the same as those for hemolysis assay. After incubation at 37 °C for 0.5 h, the samples were processed to fix with glutaraldehyde for qualitative morphological evaluation with light microscopy. A glutaraldehyde fixation method was applied for isolated cells to prevent damage that can occur during preparing smears, which are good for cells in blood. Photomicrographs of erythrocytes after incubation with pept_3 or pept_1 for comparison are presented in Figure 3.

### 2.3. Effect of Blood Plasma and Albumin on CAMP-Induced Hemolysis

The interaction with blood plasma constituents is known to play an important role in the pharmacokinetics and effectiveness of therapeutic agents. For CAMPs, the binding in plasma could decrease their effective concentration at the site of action and, thereby, their antimicrobial activity. To elucidate whether CAMP interaction with cells is affected by plasma, presumably through CAMP binding, we performed the hemolysis assay. The erythrocyte suspension was added to the same volume of PBS containing CAMP at two times the final concentration or PBS containing both CAMP and 20% autologous plasma. Calculations for extracellular hemoglobin were made based on the negative and positive control samples containing or not containing plasma.

Plasma completely inhibited the hemolytic effect of pept_2, pept_3, pept_4 and pept_5 even at the highest CAMP concentration used (100 μM final concentration) (Figure 1). As well, no cell aggregation was observed when erythrocytes were incubated with pept_3 preincubated with plasma. Pept_1 being mixed with plasma did not induce significant hemolysis at concentrations below a final concentration of 50 μM. With increasing pept_1 concentration to 100 μM the hemolysis was observed, but its level was much lower than in the absence of plasma: 15% vs. 85% (Figure 1).

Among other plasma proteins, human serum albumin (HSA) seems suitable for CAMPs to bind to it. Its molecule is highly negatively charged (–17 to –19 at physiological pH) and also prone to hydrophobic interactions. HSA, which is present in blood at a higher concentration than other proteins, constitutes approximately 60% of the total plasma protein.

**Figure 4:**
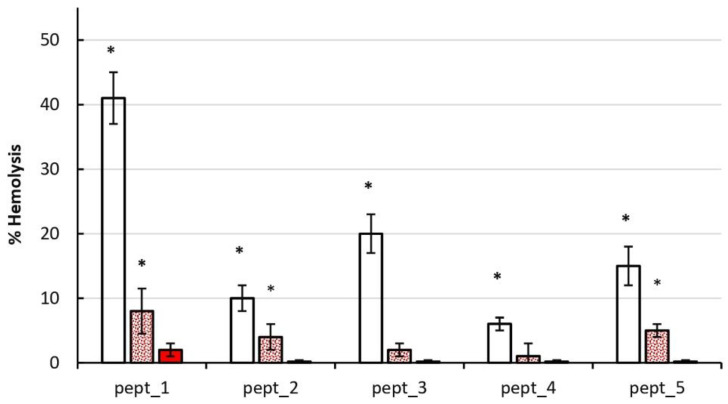
Effect of human serum albumin (HSA) on hemolytic activity of the cationic antimicrobial peptides (CAMPs) pept_1, pept_2, pept_3, pept_4 and pept_5. Erythrocytes were added to the same volume of PBS containing the peptide (open bars) or PBS containing the peptide and HSA, with a final peptide concentration of 50 μM and final HSA concentrations of 35 μM (dotted bars) and 50 μM (filled bars). Hemolysis was assayed after 0.5 h of incubation at 37 °C. Cells with Triton X-100 served as a positive control, which was set to 100%. Data shown are mean ± SD from three independent experiments using blood from three subjects, with triplicate samples in each experiment. * *p* < 0.05 compared to the corresponding positive control.

Given that the normal HSA concentration in plasma is 500–700 μM and the dilution of plasma in the final, cell-containing samples was 1:10, a final HSA concentration of 50 μM was applied. Then, 100 μM CAMP working solutions were made up in PBS or PBS containing 100 μM or 70 μM HSA to give final concentrations of 50 μM CAMP and 50 μM or 35 μM HSA. The results are shown in Figure 4. HSA dose-dependently reduced the membrane-disrupting action of CAMPs on erythrocytes, which might be attributed to CAMP binding to HSA. CAMP-induced hemolysis was completely or nearly completely (in the case of pept_1) inhibited with a higher HSA concentration of 50 μM.

### 2.4. Effect of CAMPs on Plasma Membrane Integrity of Mononuclear Cells and Neutrophils

The disruptive effect of peptides on the plasma membrane of mononuclear cells and neutrophils was evaluated by the leakage of lactate dehydrogenase (LDH) from damaged cells. Experiments were run in PBS as a medium without calcium and magnesium ions, not to favor neutrophil and monocyte activation.

Substance control was performed to determine whether CAMPs themselves could interfere with measuring LDH activity. For all CAMPs except pept_3, the absorbance values in the probes containing both CAMPs and kit’s LDH did not differ from absorbance values obtained for LDH with no added CAMPs. The presence of pept_3 at concentrations of 5 μM and 50 μM in the reaction mixture caused overestimation of LDH activity, and this peptide was not further used in the LDH assay.

**Figure 5:**
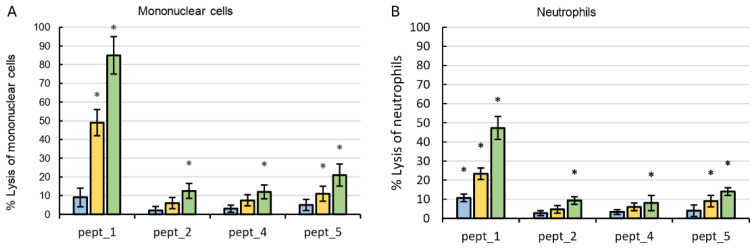
Cytolytic activity of the cationic antimicrobial peptides (CAMPs) pept_1, pept_2, pept_4 and pept_5 against mononuclear cells (**A**) and neutrophils (**B**). In each group of bars, the final peptide concentration from left to right is 5 μM, 25 μM, and 50 μM. Lysis was assayed by lactate dehydrogenase release after 0.5 h of incubation at 37 °C. Cells with Lysis solution served as a positive control, which was set to 100%. Data shown are mean ± SD from three independent experiments using blood from three subjects, with triplicate samples in each experiment. * *p* < 0.05 compared to positive control.

Aliquots of cell suspension without or with CAMPs at different concentrations were incubated at 37 °C for 0.5 h. Then, the assay steps were carried out. In each experiment, there were both mononuclear cells and neutrophils from the same healthy subject. The results are presented in Figure 5. Higher cytolytic activity against mononuclear cells and neutrophils was shown by pept_1. Overall, 50 μM pept_1 lysed about 85% of mononuclear cells and 50% of neutrophils. Compared to pept_1, pept_5 showed a lower effect, causing about 20% and 15% lysis when added at 50 µM to mononuclear cells and neutrophils, respectively. Both types of cells tended to be even more resistant to pept_2 and pept_4. According to their lytic action on mononuclear cells or neutrophils, CAMPs could be arranged as follows: pept_1 > pept_5 ≥ pept_2 ~ pept_4. This is in line with the results obtained on erythrocytes.

### 2.5. Whether CAMP-Mediated Activation of Neutrophils Could Occur

#### 2.5.1. Isolated Neutrophils

Our hemolysis results and data by others [43,44] suggest that CAMP binding in plasma can take place. On the one hand, the binding deprives CAMPs of their cytolytic action. On the other hand, CAMPs bound on, for example, protein alter its surface topography or lead to other structural alterations. Will modified plasma components cause neutrophil activation, which is involved in immune response? In an attempt to elucidate this question, we used a luminol-dependent chemiluminescence (CL) method to detect the respiratory burst of neutrophils as an indicator of their activation.

**Figure 6:**
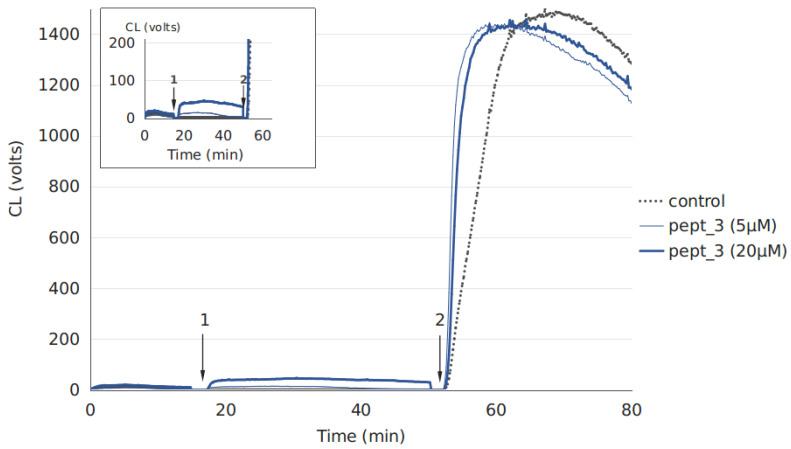
The activating effect of plasma pretreated with the cationic antimicrobial peptide (CAMP) pept_3 on neutrophils, as indicated by neutrophil respiratory burst manifested by an increase in luminol-dependent chemiluminescence (CL). Representative CL curves for neutrophils from one of three healthy volunteers. CL recording was started following the addition of luminol to the neutrophil suspension in complete Krebs–Ringer buffer medium. Arrow 1 indicates the addition of control plasma (dotted line) or plasma pretreated with pept_3 (solid lines). Plasma was pretreated at a dilution of 1:5 in PBS with pept_3 at a concentration of 50 μM or 200 μM, as it was also when performing the hemolysis assay. Final plasma dilution when measuring chemiluminescence was 1:50; final pept_3 concentration: 5 μM (thin solid line) and 20 μM (thick solid line). Arrow 2 indicates the addition of phorbol-12-myristate-13-acetate (PMA). The insert shows a magnified view of a part of the curves.

Neutrophils and plasma were obtained from the same person. Plasma diluted five times in complete Krebs–Ringer buffer solution was treated with 50 μM or 200 μM CAMP, with plasma dilution and CAMP concentrations being the same as in the samples for hemolysis assay. The final plasma content in samples for CL measurement was restricted to no higher than 2%, since plasma constituents can inhibit luminol-dependent CL due to the scavenging effect. Following the addition of luminol to neutrophils, light emission recording was started. Spontaneous cell activation, if it occurred, was allowed to complete, and then the CAMP-containing plasma or control plasma was added (Figure 6), yielding a final plasma dilution of 1:50 and a final CAMP concentration of 5 μM or 20 μM. Very low light emission levels were observed for samples with control plasma (Figure 6) and plasma containing pept_1, pept_2, pept_4 or pept_5, indicating no development of the respiratory burst. A CL increase, though relatively low, was detected only in the case of pept_3, suggesting a neutrophil respiratory burst, which results in a reactive oxygen species (ROS) production (Figure 6). The effect was dose-dependent (Figure 6).

No or low neutrophil response to the CAMP-containing plasma was not due to low cell viability, since the subsequent addition of phorbol-12-myristate-13-acetate (PMA), a standard cell stimulant, induced a high ROS production, as assessed by luminol-dependent chemiluminescence (Figure 6).

Thus, the results could mean that a portion of neutrophils in the sample was activated in response to plasma treated with pept_3.

#### 2.5.2. Neutrophils in Whole Blood

To assess whether CAMPs added to whole blood could elicit neutrophil response, the formation of neutrophil extracellular traps (NETs) was used as an indicator of neutrophil activation. NET formation encompasses both respiratory burst-dependent and respiratory burst-independent neutrophil response to different stimuli [45,46].

Hemolysis is to be avoided when investigating NET formation. Under our experimental conditions of the NET assay, hemolysis was not observed for any of the CAMPs at 100 μM, except for pept_1. Pept_1 was used at 50 μM. (For details, see the Materials and Methods section).

**Figure 7:**
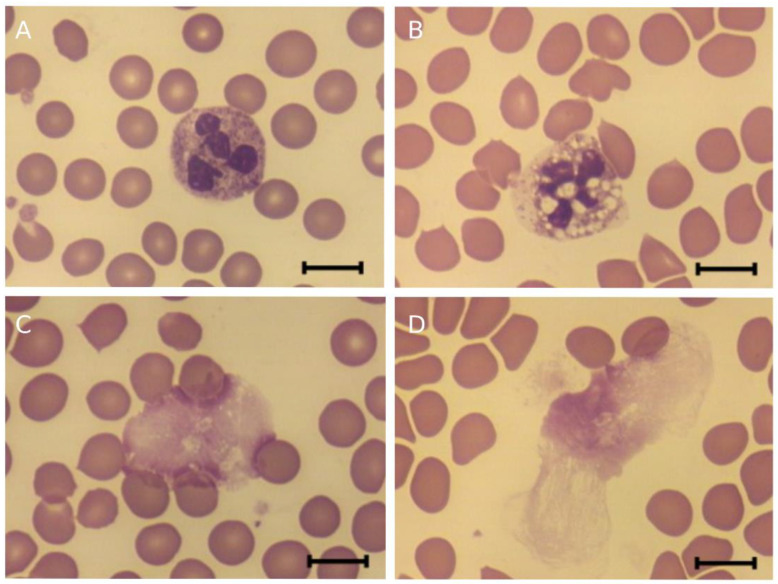
Morphology of neutrophils and neutrophil extracellular traps (NETs) in ex vivo blood after incubation (37 °C) with or without cationic antimicrobial peptides (CAMPs) in the absence or presence of neutrophil stimulation with PMA (100 nM). Concentration was 50 μM for pept_1, and 100 μM for pept_2, pept_3, pept_4 or pept_5. (**A**,**C**)—neutrophil (**A**) and NET (**C**) after 3 h blood incubation. Neutrophils (**A**) had similar morphological features, which are characteristic of resting cells, in control blood and blood supplemented with any of peptides. NETs (**C**) also did not differ in shape, size and structure in control blood and any of the peptide-exposed blood. (**B**,**D**)—neutrophil (**B**) and NET (**D**) after 3 h blood incubation, with 100 nM PMA added at the 1 h. Neutrophils (**B**) both in blood with and without peptides displayed similar morphological features characteristic of activated neutrophils (swollen shape, cytoplasmic vacuolization, and swollen nuclei). NETs (**D**) were also alike in control blood and blood supplemented with any of peptides. Shown are Romanowsky-stained smears of whole blood. Scale bar in each subfigure: 10 μm.

Microscopic analysis of neutrophils in smears prepared from blood after 3 h incubation with or without CAMPs showed no morphological features of activated cells (Figure 7A), such as, e.g., swollen shape, cytoplasmic vacuolization and swollen nuclei, which are seen in Figure 7B. Additionally, no difference in shape, size and structure of NETs was observed between control blood and any of the CAMP-exposed blood (Figure 7C). The addition of PMA (100 nM) resulted in neutrophil activation (Figure 7B), independently of whether CAMPs were present or not. PMA-induced NETs were similar in structure in blood samples both with and without CAMPs (Figure 7D).

**Figure 8:**
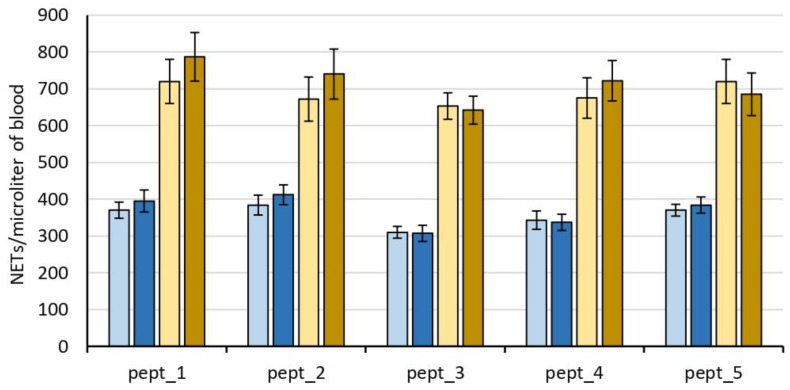
NETosis in ex vivo blood upon 3 h incubation (37 °C) with or without cationic antimicrobial peptides (CAMPs) in the absence (light and dark blue bars) or presence (light and dark ochre bars) of neutrophil stimulation with PMA (100 nM). Concentration was 50 μM for pept_1, and 100 μM for pept_2, pept_3, pept_4 or pept_5. Shown are representative data for blood from one or another healthy volunteer. In each group of bars, from left to right: blood; blood + peptide; blood + PMA; blood + peptide + PMA. No significant difference in NET concentration after 3 h incubation was revealed between control blood and blood supplemented with peptides (light and dark blue bars). NET formation in response to PMA was also similar in control blood and blood with peptides (light and dark ochre bars). The results obtained in triplicate are shown as mean ± SD.

NET concentration in the blood at time 0 h, as well as its changes upon incubation and in response to PMA, varied among persons. Its initial values were in the range of 240–320 NETs/μL, and they increased upon 3 h incubation to about 120 ± 20%. The results are shown as representative examples obtained for blood from one or another healthy volunteer (Figure 8).

No significant differences in NET concentration after 3 h incubation were revealed among control blood samples and blood samples supplemented with CAMPs (Figure 8). Neutrophils also showed a similar response to PMA both in control blood and the CAMP-exposed blood (Figure 8).

Thus, CAMPs (at the tested concentration) in whole blood did not induce NET formation as well as had no effect on NET formation stimulated with PMA.

### 2.6. Effect of CAMPs on Blood Plasma Coagulation

Leeches, like other blood-sucking organisms, synthesize compounds that are anticoagulants. Hirudin, which acts through binding to thrombin, is a well-known anticoagulant found in the leech salivary glands [47]. Could CAMPs designed based on the medicinal leech genome have an ability to affect plasma coagulation?

We examined CAMPs regarding their effects on coagulation by measuring activated partial thromboplastin time (APTT) and prothrombin time (PT) with corresponding assay kits.

It could not be ruled out that CAMPs interact with assay kit reagents. APTT and PT reagents are known to include, amongst others, phospholipids of natural or synthetic origin. Binding between CAMPs and phospholipids was demonstrated, e.g., by [48]. Our experimental results showed that the kit’s phospholipid reagent did bind our CAMPs, as indicated by the loss of their hemolytic activity in the presence of the reagent.

To overcome this problem in coagulation assays, we limited the higher CAMP concentration in plasma samples to a value at which all CAMP molecules added could be bound by plasma components and, thereby, would not further interfere with the reliable measurement of coagulation time. Our hemolysis results showed that the mixing of 100 μM pept_2, pept_3, pept_4 or pept_5 with five-fold diluted plasma apparently resulted in their complete binding, as indicated by complete inhibition of CAMP-induced hemolysis (50 μM final peptide concentration in Figure 1). As for pept_1, hemolysis was reduced to ~3%. Hence, each CAMP at 100 μM and lower concentrations in undiluted plasma is expected to be completely bound. Three CAMP concentrations were used for coagulation measurements, with the higher concentration chosen to be 100 μM.

Plasma samples (50 μL) were incubated for 15 min at room temperature with 0, 25, 50 or 100 μM CAMP, and then the coagulation assay steps were carried out according the manufacturer’s instructions.

**Figure 9:**
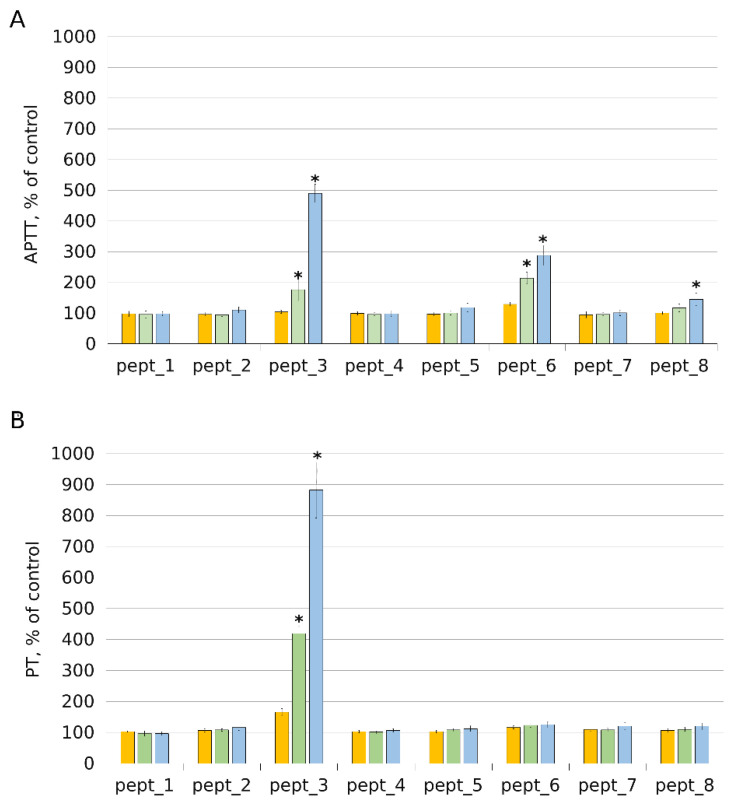
Results of plasma coagulation assays. Effect of the cationic antimicrobial peptides (CAMPs) pept_1, pept_2, pept_3, pept_4, pept_5, pept_6, pept_7 and pept_8 on activated partial thromboplastin time (APTT, (**A**)) and prothrombin time (PT, (**B**)). The coagulation kit’s normal pooled plasma was used. Before assaying for coagulation, plasma samples were pre-incubated with or without peptides for 15 min at room temperature. In each group of bars, from left to right: 25 μM, 50 μM, and 100 μM peptide concentrations in pre-incubated plasma. Plasma without peptides served as control, which was set to 100%. Data shown are mean ± SD from three independent experiments, with duplicate samples in each experiment. * *p* < 0.05 compared to control plasma.

APTT reflects the functioning of coagulation factors in the intrinsic and final common pathways of the coagulation cascade. APTT test results are shown in Figure 9A. Adding 25 μM CAMP did not lead to changes in APTT compared to APTT in control plasma samples. At higher concentrations, a considerable effect was observed for pept_3. APTT was prolonged with 50 μM pept_3 and 100 μM pept_3 by about 75% and 390%, respectively. No significant APTT change from the control value was detected for pept_1, pept_2, pept_4, and pept_5.

PT reflects the functioning of the extrinsic and common pathways. PT test results are shown in Figure 9B. As it was with APTT, PT increased when the plasma was supplemented with pept_3. The increase was dose-dependent and constituted about 165%, 320% and 780% at 25 μM, 50 μM and 100 μM pept_3, respectively, compared to control. As it was with APTT, PT was not significantly affected by pept_1, pept_2, pept_4, and pept_5.

The following experiment allowed a likely explanation for the anticoagulant effect of pept_3. Pept_3 at different concentrations (25, 50, 100, and 200 μM) was added to PBS containing 20% plasma or 20% serum (from the blood of the same volunteer), and the absorbance spectra were recorded in the wavelength range 400–800 nm using a Cary 50 Bio UV-Vis spectrophotometer (Varian, Mulgrave, Australia). Table 2 shows the optical density values (with respect to PBS) at 800 nm. No pept_3-induced changes in the transparency were observed in the serum-containing samples, whereas in the plasma-containing samples, a significant dose-dependent increase in optical density was detected at the higher pept_3 concentrations. This turbidity could be attributed to the formation of aggregates. Since serum differs from plasma only by the absence of fibrinogen, these results suggest that pept_3 in plasma could bind to fibrinogen molecules and, at relatively high concentrations, cause their aggregation. We repeated the experiment using a solution of human fibrinogen at a concentration of 0.6 mg/mL, which corresponded to that in five-times diluted plasma. The optical density of the fibrinogen solution showed a dose-related increase in response to adding pept_3, thus confirming a possible pept_3-fibrinogen complex formation (Table 2).

Fibrinogen is a coagulation factor involved in the final, common pathway of coagulation. Hence, the pept_3-induced alteration of fibrinogen structure/function should have a unidirectional effect on the results of both APTT and PT tests, which is what we observed.

Thus, of the five CAMPs pept_1, pept_2, pept_3, pept_4 and pept_5, only one of them, namely, pept_3 has a substantial impact on plasma coagulation, causing combined prolongation of APTT and PT. In addition, only pept_3 exhibited the ability to aggregate cells and proteins. To establish whether this ability is essential for strong anticoagulant activity, three CAMPs (pept_6, pept_7, and pept_8) were additionally included in the coagulation study. As with pept_1, pept_2, pept_4, and pept_5, and unlike pept_3, these CAMPs at concentrations up to 200 μM did not cause significant aggregate formation in the five-times diluted plasma, as indicated by the absence of turbidity following their addition.

Pept_6 showed an ability to prolong APTT. A 1.3-, 2.1- and 2.9-fold APTT increase over the control value was observed for pept_6 at a concentration of 25 μM, 50 μM and 100 μM, respectively (Figure 9A). However, in contrast to pept_3, pept_6 had no significant effect on PT (Figure 9B), which suggests that its influence occurs at the intrinsic pathway level, probably through binding to an intrinsic coagulation factor. A rise (by about 45%) in APTT, though much less pronounced compared to that observed with pept_3 and pept_6, was detected for pept_8 at the highest concentration used (100 μM). Pept_7 did not cause significant changes either in APTT or PT. Thus, the mechanisms of interfering with the coagulation cascade varied among different CAMPs.

## 3. Discussion

CAMPs seem to be promising new-generation antibiotics. However, their medical use is limited by various problems, including adverse effects in the human body. The strategic goal is to use CAMPs not only for topical application, but also for systemic therapy. In view of the latter, the blood biocompatibility of CAMPs should be of special attention and importance. The aim of this study was to compare the behavior of a number of synthetic short CAMPs in direct contact with blood cells and plasma. CAMPs studied were designed using bioinformatic analysis of the *Hirudo medicinalis* genome and microbiome. Their MIC values range from 10 to >100 μM.

Among five CAMPs including pept_1, pept_2, pept_3, pept_4 and pept_5, a higher hemolytic activity was observed for pept_1, with a 75–90% lysis at 100 μM (Figure 1). According to their potency to damage the erythrocyte membrane, CAMPs studied could be arranged in the following order: pept_1 > pept_3 ~ pept_5 > pept_2 ~ pept_4. This arrangement is consistent with the lysis results obtained on mononuclear cells and neutrophils (Figure 5).

Shown below are blood cell-lysing activity (1) and antibacterial activity (2) rankings, from left (most) to right (least), comparing these properties across all five CAMPs or CAMPs except pept_2 (shown in italics):

pept_1 > pept_3 ~ pept_5 > *pept_2* ~ pept_4   or   pept_1 > pept_3 ~ pept_5 > pept_4 (1)

pept_1 = *pept_2* > pept_3 > pept_5 > pept_4   or   pept_1 > pept_3 > pept_5 > pept_4 (2)

If it were not for pept_2, we could say that the two rankings are similar to some extent. An explanation for the discrepancy in the effects of pept_2 and pept_1 may be as follows. Pept_2 is an analog of pept_1, and differs from the latter only by the loss of the C-terminal Leu residue. Leu is a very hydrophobic amino acid, and, hence, its deletion/addition can change molecule’s hydrophobicity. The hydrophobicity of pept_2 was expectedly lower than that of pept_1, being 0.74 vs. 0.99 (as calculated using R package “Peptides” [49] in Table S1). Hydrophobicity is known to play an important role in the interaction of CAMPs with bacterial as well as mammalian cell membranes, enabling CAMPs to penetrate cells and induce lysis [50]. Higher hydrophobicity of CAMPs can increase their antimicrobial and hemolytic activities [51]. However, this correlation may apparently be maintained only until a certain level of hydrophobicity, beyond which the antimicrobial activity of CAMP reaches a plateau, and then decreases with increasing hydrophobicity. This was demonstrated in the studies by Chen et al. and Jiang et al., in which an increase in hydrophobicity in a series of CAMP analogs, which was achieved by systematic replacing one, two or three less hydrophobic amino acid residues with more hydrophobic one (Leu), led to higher hemolytic activity, while causing no significant changes in antimicrobial activity [52,53]. The authors have suggested the term hydrophobicity window as a peptide hydrophobicity range over which the antibacterial activity of CAMP is nearly independent of hydrophobicity, while the hemolytic activity continues to depend on it. We hypothesize that this phenomenon may refer to pept_1 and pept_2. Pept_2, which was less hydrophobic than pept_1, was also less cytolytic, with antibacterial activity being the same as for pept_1.

In addition to causing hemolysis, pept_3 also exhibited an ability to cause cell aggregation (Figure 2 and Figure 3), and appeared able not only to bind to, but also to aggregate proteins as exemplified for human fibrinogen (Table 2). The CL increase, though small, following addition of pept_3-containing plasma to isolated neutrophils (Figure 6) might be interpreted as the neutrophil activation in response to pept_3-induced aggregates of plasma components. In whole blood, no neutrophil response (as assessed by NET formation) was observed either for pept_3 or other CAMPs at the chosen concentration (Figure 7 and Figure 8).

Overall, the results on CAMP cytolytic activity against blood cells showed that pept_2 possessing a higher antibacterial potency was, on the contrary, relatively less cytolytic.

We applied our results on cytotoxicity to make a comparative evaluation of the resistance of different blood cells to lysis by CAMPs. Based on the number of cells and CAMP molecules in the sample in the hemolysis assay and LDH assay, we estimated, albeit roughly, how many CAMP molecules per cell were needed to achieve the same degree of lysis in different cell types. For example, 50% lysis in erythrocytes, mononuclear cells or neutrophils was achieved with pept_1 at a concentration of 65, 25 and 50 μM, respectively, which corresponded to 2.6 × 10^9^, 50 × 10^9^ and 100 × 10^9^ peptide molecules per cell or approximately 20 × 10^6^, 430 × 10^6^ and 500 × 10^6^ peptide molecules per square micrometer of cell surface. For reference, the surface area of erythrocytes is about 135 μm^2^, that of lymphocytes (which make up the majority of the mononuclear cell population) about 115 μm^2^, and that of neutrophils about 200 μm^2^. Thus, it may be suggested that the membrane of erythrocytes is less resistant to exposure to CAMPs than the membrane of leukocytes, among which neutrophils are more resistant. Considering that the damaging action of CAMPs on cells is mediated by electrostatic interaction, one reason for a higher sensitivity of erythrocyte plasma membrane to CAMPs may be its higher electronegativity. The zeta potential of human erythrocytes at pH 7.4 is about −32 mV, and that of mononuclear cells about −22 mV [54]. A greater negative surface charge promotes more active binding of CAMPs.

Plasma binding of drug products is known to be not uncommon. Hence, biocompatibility studies of compounds considered as potential therapeutic agents should include experiments in a plasma-containing medium. Our results showed that when erythrocytes were added to CAMPs in PBS supplemented with plasma, hemolysis was not detected, or it was considerably repressed compared to that in the absence of plasma (Figure 1). This could be attributed to CAMP binding in plasma, with an affinity sufficient to prevent CAMP translocation to cells.

HSA is known to have a high binding capacity for numerous endogenous and exogenous compounds. Our CAMPs are, probably, not the exception in this regard, which is indirectly reflected by reduced hemolysis in the presence of HSA (Figure 4). The binding of short CAMPs to human or bovine serum albumin has been reported by [43,44]. CAMPs bound to albumin were shown to lose their antibacterial activity [43].

Though HSA is abundant in plasma and can efficiently bind CAMPs, the latter being added to plasma may also be bound by other proteins. This was demonstrated by our plasma coagulation experiments indicating the binding of CAMPs to coagulation factors. Pept_6 produced the marked prolongation of APTT and no significant effect on PT, suggesting the peptide interacts with a factor (or a few factors) involved in the intrinsic coagulation pathway, with the exception of factors involved in the final common pathway. The addition of pept_3 to plasma considerably prolonged both APTT and PT. It might be proposed that pept_3 binds to coagulation factors of both intrinsic and extrinsic pathways. However, given our data indicating the ability of pept_3 to bind to fibrinogen (Table 2), one could also speculate that the combined effect on APTT and PT is possible solely due to pept_3 binding to fibrinogen, a coagulation factor belonging to the common coagulation pathway. Thus, pept_3 and pept_6 exhibited dual antimicrobial and anticoagulating properties. Other CAMPs under study had no effect or exerted mild prolongation of plasma coagulation time.

## 4. Materials and Methods

### 4.1. Reagents

Phosphate buffered saline (PBS) tablets; Histopaque-1077 (density: 1.077 g/mL) and Histopaque-1119 (density: 1.119 g/mL) solutions; calcium chloride (CaCl_2_), sodium bicarbonate (NaHCO_3_), D-glucose, Krebs–Ringer bicarbonate buffer powder with 1800 mg/L glucose and no CaCl_2_ and NaHCO_3_, dimethyl sulfoxide (DMSO), human fibrinogen, melittin, luminol, and phorbol 12-myristate 13-acetate (PMA) were purchased from Sigma-Aldrich (Merck Company Inc., Kenilworth, NJ, USA).

Synthesis of peptides was carried out by solid-phase peptide synthesis in a Liberty Blue automated microwave peptide synthesizer (CEM, Stallings, NC, USA) by using Rink Amide NovaGel Novabiochem (0.69 mM/g) as the solid phase [38]. Fmoc-protected amino acid derivatives from Sigma Aldrich were applied in the synthesis. Preparative purification of synthesized products was carried out by reversed-phase chromatography in H_2_O/ACN gradient. Peptide purity was verified by HPLC-MS. ZORBAX SB-C18 chromatography columns (Agilent Technologies, Santa Clara, CA, USA) were used for quantitative chromatographic analysis and confirmation of peptide purity. All peptides were >95% pure.

### 4.2. Peptide Solutions

First, 2 mM stock solutions of CAMPs were prepared in PBS, except for the CAMPs pept_1 and pept_2. Pept_1 is poorly soluble in PBS. Its 2 mM solution was made up using PBS containing 4% DMSO (PBS/DMSO), which gave a final DMSO concentration not higher than 0.2% in any of experiments. Of particular interest was to compare pept_2 to pept_1, which only differs from pept_2 by one additional amino acid residue at the C-terminus. Hence, the stock solution of pept_2 was prepared similarly to that of pept_1. DMSO has a potential to cause cell lysis. Therefore, negative controls containing relevant concentrations of DMSO were run for pept_1 and pept_2, and DMSO contribution was eliminated by subtracting the measured value in the negative control samples from that in the test samples. A correct accounting of the DMSO contribution was confirmed by consistency between the results obtained for pept_2 dissolved in PBS and pept_2 dissolved in PBS/DMSO.

### 4.3. Blood Collection

Peripheral blood from healthy volunteers was collected in vacutainer tubes (BD, Mumbai, India). All the volunteers (*n* = 7) agreed based on the informed consent to participate in the research.

### 4.4. Isolation of Blood Mononuclear Cells and Neutrophils

Mononuclear cells and neutrophils were isolated from EDTA-anticoagulated blood by density gradient centrifugation using Histopaque-1119 plus Histopaque-1077 density solutions according to the manufacturer’s instructions. The ring of mononuclear cells was taken from the plasma/Histopaque-1077 interface, and the ring of neutrophils—from the Histopaque-1077/Histopaque-1119 interface.

Harvested mononuclear cells may contain contaminant platelets. To get rid of them, low speed and short centrifugation (120× *g*, 5 min) was used for washing the cells. To reduce platelet activation allowing them to adhere to other cells, a buffer free of Ca^2+^ and Mg^2+^ is recommended. We used PBS supplemented with 1800 mg/mL glucose as a cell nutrient. The washing was repeated twice, and mononuclear cells were suspended in the desired buffer medium.

Harvested neutrophils were washed twice with PBS plus glucose by centrifugation for 15 min at 400× *g*. The residual contaminant erythrocytes were removed by hypotonic lysis after the first washing step. Neutrophils were finally suspended in the desired buffer medium.

Cell viability assessment was performed with trypan blue staining in a Goryaev counting chamber (MiniMed, Bryansk, Russia), with viability being about 99%.

### 4.5. Isolation of Erythrocytes

Erythrocytes were isolated from EDTA-anticoagulated blood. The blood was centrifuged for 10 min at 120× *g*. A volume of 0.5 mL of settled erythrocytes was drawn from the middle of the erythrocyte column height and transferred for washing in 12 mL of PBS in a centrifuge tube. The cells were centrifuged for 10 min at 120× *g*. The washing procedure was repeated twice more.

### 4.6. Procedure for Obtaining Plasma and Serum

To obtain plasma, blood was collected into EDTA-containing vacutainer tubes and centrifuged at 400× *g* for 15 min. To obtain serum, blood was collected into vacutainer tubes containing a clot activator and serum gel separator, allowed to clot for 30 min, and centrifuged at 400× *g* for 15 min. The upper two thirds of plasma or serum layer were additionally centrifuged at 3000× *g* for 20 min to remove all cell and platelet contamination.

### 4.7. Cytolytic Activity against Erythrocytes

The hemolytic activity of CAMPs was determined using a hemolysis assay measuring hemoglobin released from damaged cells. A working erythrocyte suspension (in PBS) was prepared so that its ten-fold dilution gave an optical density of 0.4 at 800 nm. This working suspension corresponded to a cell density of about 30 × 10^6^ cells/mL. Aliquots of 125 μL of CAMP solutions of different concentrations were added to Eppendorf tubes. The medium used for preparing CAMP solutions served for negative control, and the same medium, additionally supplemented with 0.2% Triton X-100, served for positive control. Next, 125 μL of erythrocytes were added to each Eppendorf tube. Following incubation (with slight shaking) for 0.5 h at 37 °C, the samples were centrifuged at 1000× *g* for 10 min. The supernatant (200 μL) was transferred to the wells of a microplate, and the absorbance of hemoglobin was measured by a Thermo Electron Multiskan Ascent microplate reader (Thermo Fisher Scientific Inc., Waltham, MA, USA), with a test wavelength of 414 nm as well as 540 nm and a background wavelength of 690 nm. The hemolysis percentage was calculated using the following equation: Hemolysis (%) = {(sample absorbance − negative control)/(positive control − negative control)} × 100.

### 4.8. Cytolytic Activity against Mononuclear Cells and Neutrophils

The cytolytic activity of CAMPs against mononuclear cells and neutrophils was determined using a lactate dehydrogenase (LDH) assay measuring LDH released upon cell lysis. Working cell suspensions in PBS supplemented with 1800 mg/mL glucose were adjusted to an optical density of 0.12 at 800 nm, which corresponded to a cell density of about 600 × 10^3^ cells/mL. LDH assay was performed using a Cytotoxicity Detection Kit^PLUS^ (LDH) (Roche Diagnostics, Mannheim, Germany) according to the manufacturer’s protocol. Then, 50 μL aliquots of CAMP solutions of different concentrations were placed in the wells of a microplate. The medium used for preparing CAMP solutions and the kit’s Lysis solution served for negative and positive controls, respectively. Next, 50 μL of cell suspension were added to each well and incubated for 0.5 h at 37 °C, after which LDH enzymatic reaction was triggered by adding 100 μL of the kit’s Reaction mixture. After the color had developed, the reaction was stopped by adding 50 μL of the kit’s Stop solution. The plate was read at 492 nm and 690 nm as background. The cytolysis percentage was calculated using the following equation: Cytolysis (%) = {(sample absorbance − negative control)/(positive control − negative control)} × 100.

### 4.9. Neutrophil Extracellular Trap (NET) Assay

EDTA-anticoagulated capillary blood was obtained via a finger prick. Blood aliquots of 20 μL were placed in 1.5 mL Eppendorf tubes and mixed with 2 μL of a CAMP solution (or 2 μL of PBS in controls). The samples were incubated for 3 h. After the first 1 h, PMA (100 nM final concentration) was added to a part of them, and incubation continued for a further 2 h. Cell settling was prevented by continuous gentle turning the tubes up and down using a Multi Bio RS-24 rotator (Biosan, Riga, Latvia). The blood was retained at the tube bottom by surface tension.

Hemolysis should be avoided when investigating NET formation. Preliminary experiments were conducted to ensure that hemolysis would not occur for the duration of 3 h at a chosen CAMP concentration of 100 μM. The same volume proportion of CAMP solution to blood and the same mode of mixing, as described above, were maintained with a single discrepancy in the final sample volume that was larger (500 μL in a 500 μL-Eppendorf tube) to have plasma in an amount sufficient for the spectrophotometric measurement of hemoglobin. Hemolysis was not observed for any of CAMPs at 100 μM, except for pept_1. Pept_1 was used at 50 μM.

NET counts were performed using standardized blood smears stained by Romanowsky method. The smears were examined using a Motic B microscope (Motic, Xiamen, China). Quantitative assessment of NETs was performed as described previously [55]. NETs among 300–500 leukocytes were counted in the middle third part of the smear, and the NETs-to-leukocyte percentage ratio was calculated. Based on counting results, the number of NETs in 1 μL of blood was calculated. In each experiment, two parallel samples were prepared and three parallel smears were taken from each at each observation point.

### 4.10. Morphological Observation of Isolated Erythrocytes

Samples for morphological observation of erythrocytes were the same as those for hemolysis assay. After incubation at 37 °C for 0.5 h, the samples were centrifuged at 100× *g* for 5 min, following which the supernatant was removed and the cells fixed by resuspending them in 0.5 mL of 1% glutaraldehyde solution in PBS. Then, erythrocytes were left for 3 h at room temperature, with regular mixing. The fixed cells were stored at 4 °C until use. The supernatant over settled cells was withdrawn, and a cell aliquot was placed in a small workspace cut in the piece of scotch tape put on a microscope slide. Images were examined using a Motic B light microscope (Motic, Xiamen, China).

### 4.11. Chemiluminescence Assay

Chemiluminescence study was conducted at 37 °C with a Lum-1200 chemiluminometer (DISoft Llc, Moscow, Russia) measuring the intensity of emitted light in volts. Assays were performed with the following components in a total volume of 500 μL:435 μL of neutrophil suspension (final, 0.5 × 10^6^ cells/mL) in complete Krebs–Ringer buffer solution, 10 μL of luminol (final, 200 μM), 50 μL of five-fold diluted (in the above buffer solution) autologous plasma containing or not containing CAMP at a desired concentration, 5 μL of PMA (final, 160 nM).

Neutrophils were placed in chemiluminometer cuvettes and equilibrated at 37 °C in the cuvette holder, after which luminol was added to each sample and the light emission was continuously recorded. In desired time intervals, plasma mixed with CAMP (or control plasma) and then PMA were added.

### 4.12. Coagulation Assay

The plasma coagulation tests were performed using an APG4-03-Pkh hemostasis analyzer (EMCO Llc, Moscow, Russia) and MLT-APTT and MLT-THROMBOPLASTIN reagent kits (MLT Llc, Moscow, Russia) according to the manufacturer’s instructions. Testing was performed on Plasma N (normal), a mixed pool of citrate anticoagulated plasma obtained from healthy donors, manufactured by RENAM Rpd, Moscow, Russia. Fifty microliter plasma samples supplemented with CAMPs at different concentrations (or with PBS as control) were pre-incubated at room temperature for 15 min. Afterward, in case of measuring activated partial thromboplastin time (APTT), 50 μL of APTT Reagent were added. The samples were warmed at 37 °C for 3 min, and 50 μL of Calcium Chloride Solution were added to initiate fibrin clot formation. For measuring prothrombin time (PT), pre-incubated plasma samples were warmed at 37 °C for 1 min, and fibrin clot formation was triggered by adding 100 μL of Thromboplastin Reagent.

### 4.13. Minimum Inhibitory Concentration (MIC) Determination

*Escherichia coli* str. K12 substr. MG1655 (Invitrogen) and *Bacilus subtilis* str. 168HT (Vavilov Institute of General Genetics, Russian Academy of Sciences, Moscow, Russia) were grown in Luria–Bertani broth (Difco, BD Difco, Thermo Fisher Scientific Inc., Waltham, MA, USA) according to standard procedure. Bacteria were stored with 10% glycerol at −70 °C until use. MIC determination was carried out by growing bacteria in 96-well microtitration plates in the presence of a two-fold dilution series of the peptide using the standard microtiter dilution method [56]. Briefly, single bacterial clones were cultured overnight in Mueller–Hinton broth (MHB, BD Difco, Thermo Fisher Scientific Inc., Waltham, MA, USA) at 150 rpm and 37 °C, then transferred to fresh MHB and cultured until the logarithmic growth phase. Bacteria were then diluted to 106 CFU/mL in MHB. The peptides were dissolved and diluted in deionized water. Aliquots of 50 μL of the bacterial culture suspension were placed into microtiter plate wells, followed by the addition of 50 μL of the diluted peptide (0.5–128 μg/mL, final concentrations). After incubation at 37 °C for 24 h, the antimicrobial susceptibility was estimated by turbidity measurement at 560 nm using a microplate reader. The MIC was determined as the lowest peptide concentration at which bacteria failed to grow. Bacteria treated with melittin served as a positive control, and untreated bacteria (no peptide) served as a negative control. Data are presented as the mean value from three independent experiments with triplicates in each experiment.

### 4.14. Statistical Analysis

Data are expressed as mean ± standard deviation of three independent experiments using blood of three persons, with triplicate or duplicate samples in each experiment. Due to the non-Gaussian distribution of the means, the nonparametric Wilcoxon–Mann–Whitney test was used to determine a significant difference between the control samples and the others. A *p*-value < 0.05 was considered statistically significant.

## 5. Conclusions

A number of novel synthetic short CAMPs were compared regarding their cytolytic action on human blood cells, and their effect on plasma coagulation. One of them, the CAMP pept_2, was found to combine a higher bactericidal activity, a lower cytolytic activity against blood cells and no effect on plasma coagulation. Blood plasma and albumin inhibited CAMP-induced hemolysis, presumably due to CAMP binding to albumin and other plasma components, with the affinity preventing translocation onto cells. The results clearly indicate that the assessment of CAMP biocompatibility should include not only evaluation of their cytotoxicity, but also a study of their interaction with blood plasma. Bound peptides could alter the protein function/activity. Plasma binding reduces the effective concentration of CAMPs available to damage cells and, at the same time, to combat bacteria.

## Figures and Tables

**Table 1:** Cationic antimicrobial peptides (CAMPs) used in this study and their characteristics.

Peptide Name	Amino Acid Sequence	Mol. Mass, Da	Charge (at Physiol. pH)	MIC, μM (E. coli)	References
pept_1	FRIMRILRVLKL	1558.05	+4	10	[38]
pept_2	FRIMRILRVLK	1444.89	+4	10	present study
pept_3	RWRLVCFLCRRKKV	1863.36	+6	17	[38]
pept_4	KFKKVIWKSFL	1423.81	+4	90	[38]
pept_5	RPILIRVRRIRVI	1660.13	+5	77	[38]
pept_6	RLKRFKRVALRREKTARNFRSIVS	2988.61	+9	>100	[40]
pept_7	FLIGKAIKRKFCLRSVWNA	2250.81	+5	14.3	[39]
pept_8	RAVIYKIPYNAIASRWIIAPKKC	2675.31	+5	24	[40]

**Table 2:** The cationic antimicrobial peptide (CAMP) pept_3 causes aggregation of fibrinogen, as indicated by an increase in optical density at 800 nm in the samples containing human fibrinogen or plasma in contrast to no increase in the samples containing serum having no fibrinogen. Data shown are mean ± SD from three independent experiments.

	Optical Density at 800 nm		
pept_3, μM	PBS Containing 20% Plasma	PBS Containing 20% Serum	PBS Containing Fibrinogen (0.6 mg/mL)
0	0.03 ± 0.01	0.03 ± 0.01	0.00
50	0.03 ± 0.01	0.03 ± 0.01	0.19 ± 0.01
100	0.11 ± 0.02	0.03 ± 0.01	0.22 ± 0.01
200	0.69 ± 0.06	0.03 ± 0.01	0.26 ± 0.02

## Data Availability

The data used to support the findings of this study are included within the article. Additional information may be obtained from the corresponding author upon request.

## Supplementary Materials

The following supporting information can be downloaded at: https://www.mdpi.com/article/10.3390/molecules27185848/s1, Table S1: Physical-chemical properties of cationic antimicrobial peptides (CAMPs) used in this study. Calculations were made using R package “Peptides”.

## Sample Availability

The samples are not available from the authors.
