# Supplementary material for: Effects of Medicinal Leech-Related Cationic Antimicrobial Peptides on Human Blood Cells and Plasma
Source: Molecules. 2022 Sep 9;27(18):5848. doi: 10.3390/molecules27185848 (PMC9503446; doi:10.3390/molecules27185848)
Supplement: Supplementary file 1 [file molecules-27-05848-s001.zip › molecules-1878101-supplementary.pdf]

Table S1. Physical-chemical properties of cationic antimicrobial peptides (CAMPs) used in this study. Calculations were made using R package "Peptides" [49].

Peptide name	Amino acid sequence	Hydro-phobicity	Aliphatic index	Net charge (at physiol. pH)	pI
pept_1	FRIMRILRVLKL	0.99	186.67	+4	12.8
pept_2	FRIMRILRVLK	0.74	168.19	+4	12.8
pept_3	RWRLVCFLCRRKKV	–0,21	97.14	+6	12.0
pept_4	KFKKVIWKSFL	0.07	97.27	+4	11.0
pept_5	RPILIRVRRIRVI	0.47	194.62	+5	13.1
pept_6	RLKRFKRVALRREKTARNFRSIVS	–0.95	81.25	+9	12.9
pept_7	FLIGKAIKRKFCLRSVWNA	0.33	107.8	+5	11.6
pept_8	RAVIYKIPYNAIASRWIIAPKKC	0.21	114.78	+5	10.6
